# LINKING PLASMA PROTEOMICS TO BRAIN ACCELERATED/RESILIENT AGING IN THE ATHEROSCLEROSIS RISK IN COMMUNITY STUDY

**DOI:** 10.1093/geroni/igad104.2769

**Published:** 2023-12-21

**Authors:** Ramon Casanova, Jamie Justice, Keenan Walker, Andrea Anderson, Ryan Barnard, Tim Hughes, Stephen Kritchevsky, Lynne Wagenknecht

**Affiliations:** Wake Forest University, Winston Salem, North Carolina, United States; Wake Forest University School of Medicine, Winston-Salem, North Carolina, United States; National Institute on Aging, National Institutes of Health, Baltimore, Maryland, United States; Wake Forest University School of Medicine, Winston-Salem, North Carolina, United States; Wake Forest University School of Medicine, Winston-Salem, North Carolina, United States; Wake Forest University School of Medicine, Winston-Salem, North Carolina, United States; Wake Forest University School of Medicine, Winston-Salem, North Carolina, United States; Atrium Health/Wake Forest Baptist, Winston-Salem, North Carolina, United States

## Abstract

Machine learning models are increasingly being used to estimate ‘brain age’ from neuroimaging data. The gap between chronological age and the estimated brain age (BAG) is potentially a measure of accelerated/resilient brain aging, and we estimated BAG based on an elastic net regression approach. Here, we report associations between this brain age measure and plasma protein levels (measured using an aptamer-based proteomic platform) in the Atherosclerosis Risk in Communities (ARIC) Study to determine whether BAG was associated with proteins linked to biologic aging. We used brain MRI scans from 1507 ARIC visit 5 participants: 938 were Cognitively Normal (CN), 495 had mild cognitive impairment (MCI) and 74 had dementia. The proteomic dataset contains 4877 plasma proteins in these same individuals. We fitted univariate linear regression models adjusting for age, race, smoking, education, sex, diabetes, hypertension and intra-cranial volume. A Bonferroni correction was applied to account for multiple testing to maintain an analysis-wise α=0.05. In total 31 proteins were significantly associated with BAG. Among these SEVP1, GDF-15, pleiotrophin, MMP7, Natriuretic-peptides and Hsp70 were found to be associated with accelerated BAG, whereas EGF-receptor, mast/stem-cell-growth-factor-receptor (KIT), Coagulation-factor-VII and cGMP-dependent-protein-kinase-1 were associated to resilient BAG. The analysis based only on CN participants produced 3 significant proteins: retinoblastoma-2 and SEVP1, which were associated with accelerated BAG and Coagulation-factor-VII, which was associated to resilient BAG. These results suggest circulating proteins implicated in biological aging, cellular senescence, angiogenesis and coagulation are associated with a neuroimaging measure of accelerated brain aging.

